# Supplementary figures and images for: CStone: A de novo transcriptome assembler for short-read data that identifies non-chimeric contigs based on underlying graph structure
Source: PLoS Comput Biol. 2021 Nov 23;17(11):e1009631. doi: 10.1371/journal.pcbi.1009631 (PMC8651127; doi:10.1371/journal.pcbi.1009631)

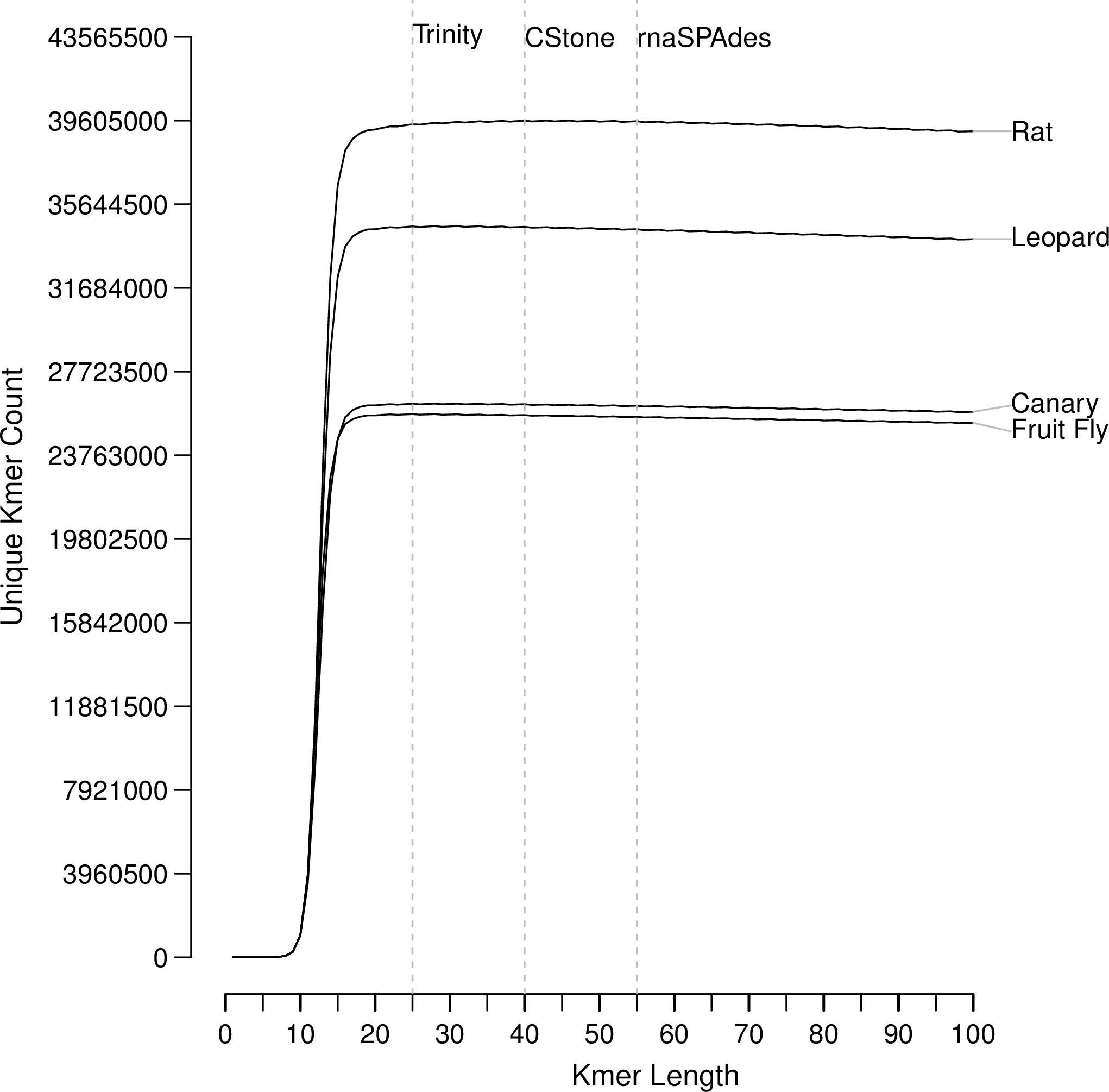

Supplement: S1 Fig — Reads were simulated from the four species (indicated on right) as described under the “Demonstration” heading of the Design and Implementation section of the manuscript. Above kmer size of 18 little difference is observed in the number of unique kmers extracted. Kmer sizes from 1 to 18 show a marked increase as kmer frequency as size is incremented in steps of 1. This indicates that for these small kmers, shared kmers by chance (or kmer collisions) between different gene families and gene regions are more likely. For example, for kmer sizes of 4 there are only 256 unique permutations to describe the entire read dataset. Assemblies using kmers of this size would produce spurious sets of contigs that are highly chimeric. The three assemblers used in this study were Trinity, CStone and rnaSPAdes and have default kmer sizes of 25, 40 and 55 respectively, as indicated with the dashed vertical lines. (TIF) [file pcbi.1009631.s001.tif]

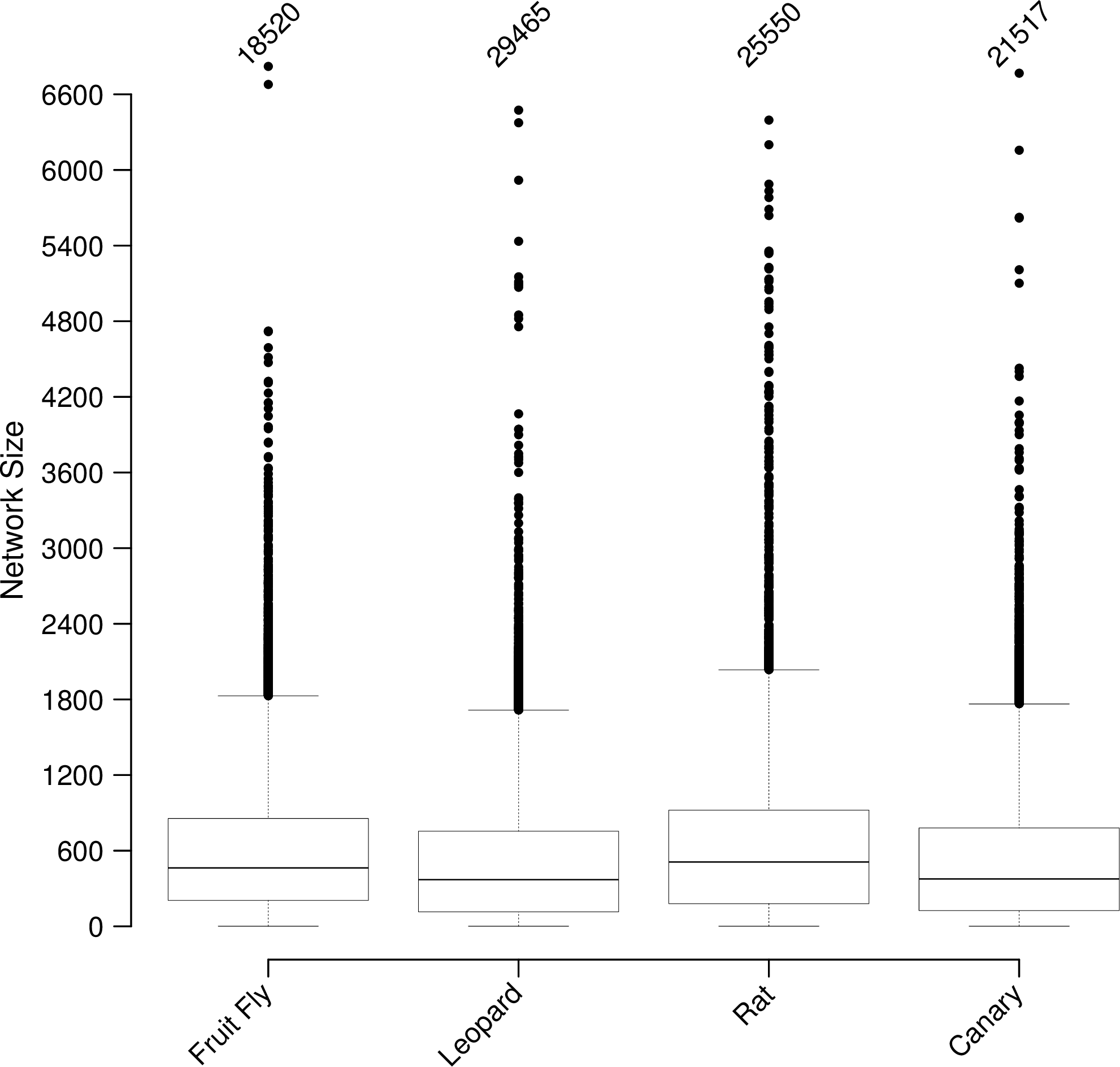

Supplement: S2 Fig — Following the edge connection step within CStone groups of connected edges, i.e. graphs, are extracted prior to the software identifying contigs. The size range of these networks (box and whiskers) and total numbers (top) are indicated for each of the simulated datasets (same as for S1 Fig) from the four species used within this study. Boxes represent the sizes falling within the inter quartile ranges. The median is shown within each box. Whiskers extend to the furthest data point that is within 1.5 times the inter quartile range and points beyond this are outliers (black circles). (TIF) [file pcbi.1009631.s002.tif]

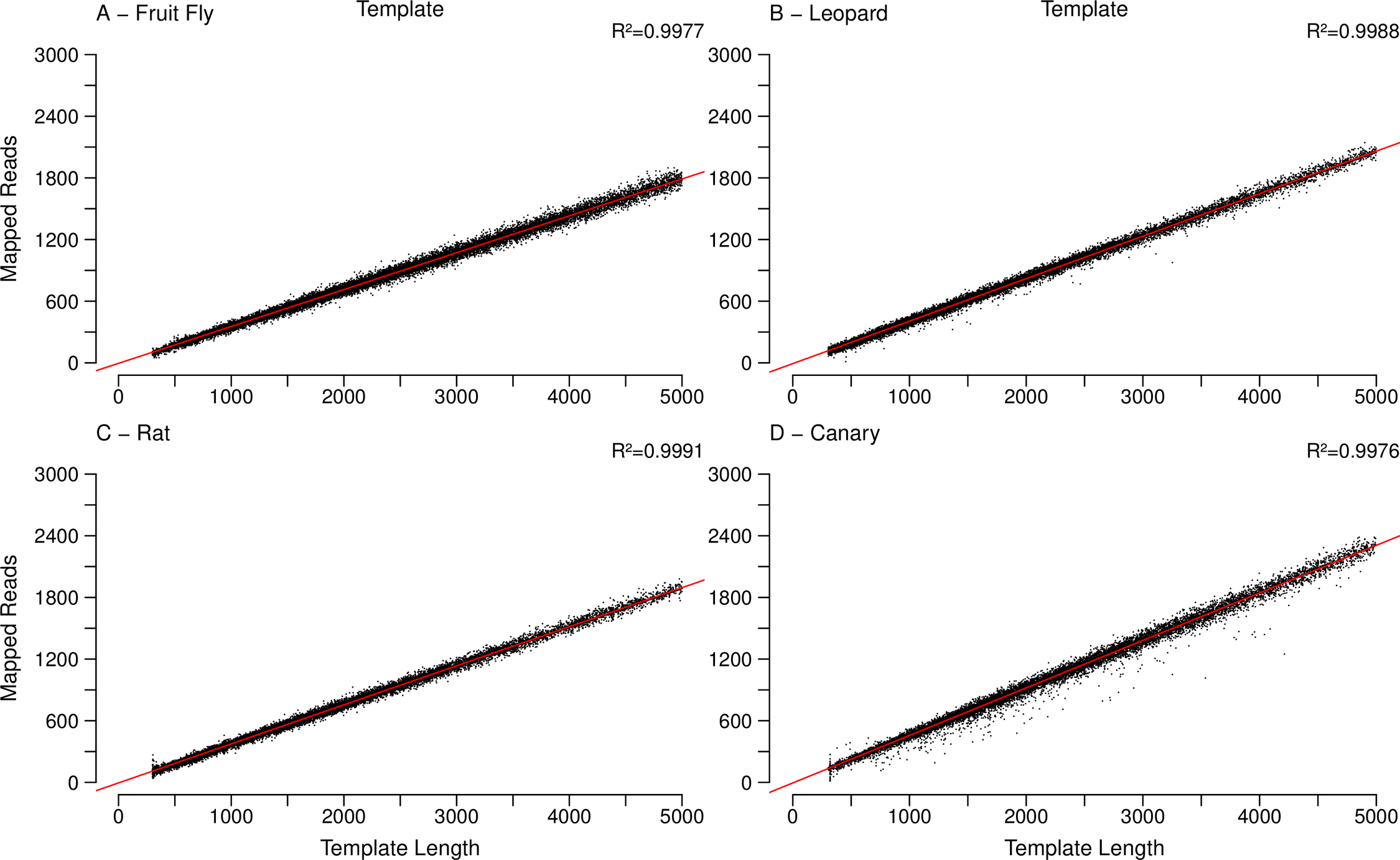

Supplement: S3 Fig — Simulated reads containing no sequencing error, and distributed evenly across all transcripts, were mapped back to the cDNA transcripts from which they were generated in order to visualize the expected linear relationship between mapped read count and cDNA reference transcript length. Subsequent contigs assembled from these reads should also reflect this linear relationship, if not it is the first indication of poor quality assemblies. (TIF) [file pcbi.1009631.s003.tif]

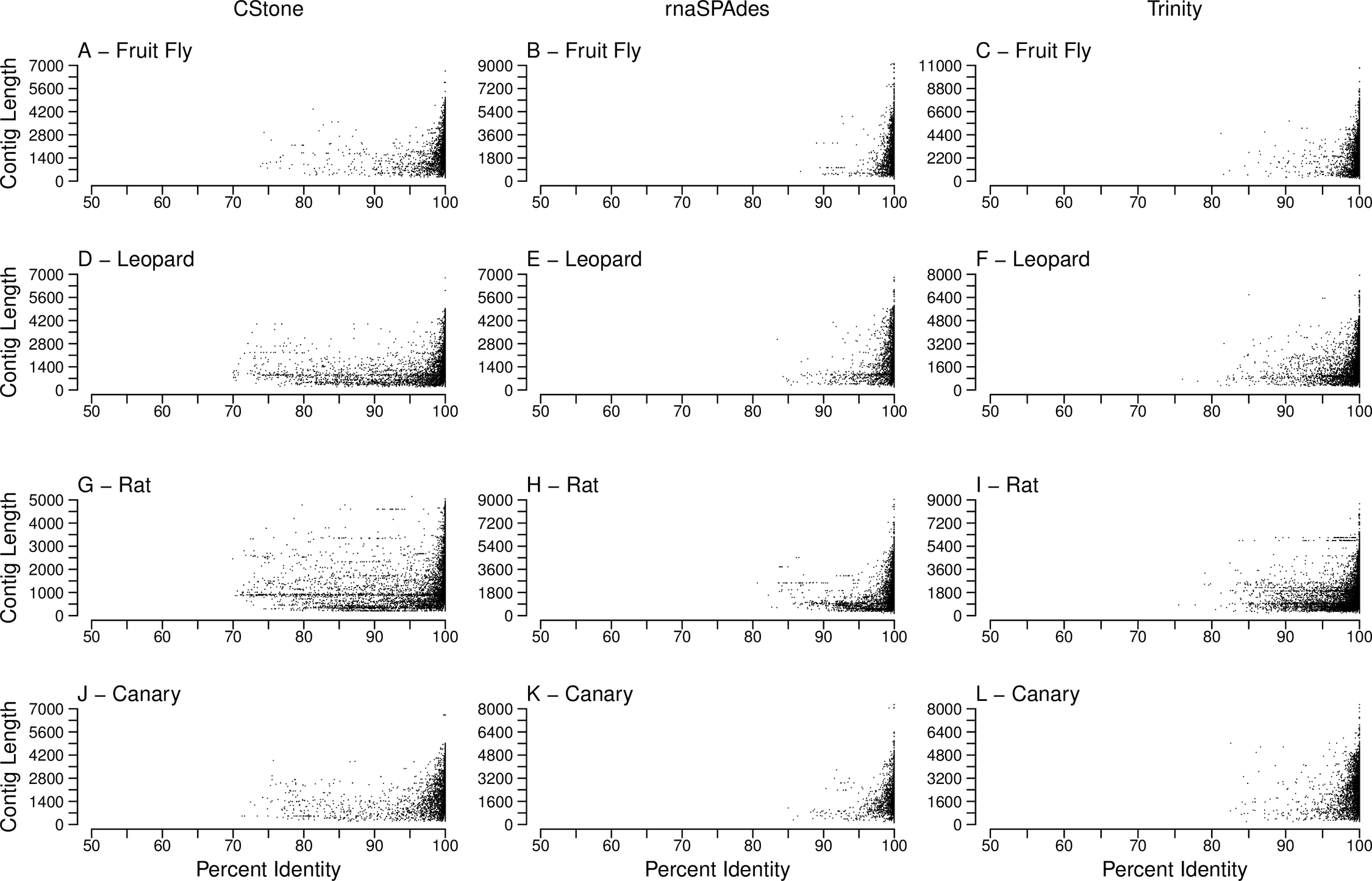

Supplement: S4 Fig — These plots are a visualization of the sequence identities presented in Table 6. (TIF) [file pcbi.1009631.s004.tif]
