# Supplementary material for: CStone: A de novo transcriptome assembler for short-read data that identifies non-chimeric contigs based on underlying graph structure
Source: PLoS Comput Biol. 2021 Nov 23;17(11):e1009631. doi: 10.1371/journal.pcbi.1009631 (PMC8651127; doi:10.1371/journal.pcbi.1009631)
Supplement: S1 Table — (DOCX) [file pcbi.1009631.s005.docx]

**S1 Tabe: Virus reference genomes from NCBI that matched with contigs representing whole-adult 1 using megablast.**

| **Assembler** | **Contig ID** | **Contig**  **Length** | **Matched**  **Species** | **Accession** | **Length Of Aligned Region** | **Percent Identity** |
| --- | --- | --- | --- | --- | --- | --- |
| CStone | 17775 | 1842 | Drosophila immigrans Nora virus | NC_024488.1 | 1864 | 77.20 |
|  | 17776 | 1842 | Nora virus | NC_007919.3 | 1842 | 97.07 |
|  | 19552 | 1560 | Lymantria dispar multiple nucleopolyhedrovirus | NC_001973.1 | 927 | 75.73 |
|  | 17790 | 2317 | Tokyovirus A1 | NC_030230.1 | 110 | 80.00 |
|  | 1426 | 3479 | Choristoneura fumiferana granulovirus | NC_008168.1 | 95 | 92.63 |
|  | 1993 | 867 | Diadromus pulchellus toursvirus | NC_011335.1 | 64 | 90.63 |
|  | 645 | 362 | Mycobacterium phage Wonder | NC_042058.1 | 64 | 87.50 |
|  | 34917 | 679 | Cyprinid herpesvirus 2 | NC_019495.1 | 44 | 88.64 |
|  | 36826 | 685 | Gordonia phage Adgers | NC_048695.1 | 39 | 94.87 |
|  | 50246 | 324 | Cynomolgus cytomegalovirus | NC_033176.1 | 38 | 94.74 |
|  | 20479 | 529 | Enterobacteria phage P2 | NC_041848.1 | 33 | 100.00 |
|  | 55163 | 271 | Pandoravirus salinus | NC_022098.1 | 31 | 96.77 |
|  | 1383 | 1009 | Aeribacillus phage AP45 | NC_048651.1 | 31 | 100.00 |
|  | 35124 | 340 | Adoxophyes honmai nucleopolyhedrovirus | NC_004690.1 | 28 | 100.00 |
|  | 12552 | 867 | Trichoplusia ni single nucleopolyhedrovirus | NC_007383.1 | 28 | 100.00 |
|  | 13009 | 1822 | Chrysochromulina ericina virus | NC_028094.1 | 28 | 100.00 |
|  | 45858 | 236 | Pandoravirus macleodensis | NC_037665.1 | 28 | 100.00 |
|  | 47783 | 275 | Mythimna unipuncta nucleopolyhedrovirus | NC_043530.1 | 28 | 100.00 |
|  | 24441 | 1543 | Vibrio phage VAP7 | NC_048765.1 | 28 | 100.00 |
| rnaSPAdes | 404 | 7548 | Nora virus | NC_007919.3 | 7546 | 97.12 |
|  | 8749 | 1654 | Lymantria dispar multiple nucleopolyhedrovirus | NC_001973.1 | 911 | 75.85 |
|  | 11377 | 1187 | Escherichia phage RCS47 | NC_042128.1 | 264 | 90.15 |
|  | 34446 | 193 | BeAn 58058 virus | NC_032111.1 | 195 | 87.69 |
|  | 29381 | 229 | Shigella phage Shfl2 | NC_015457.1 | 181 | 87.29 |
|  | 45116 | 165 | Pseudomonas phage | NC_030909.1 | 165 | 95.76 |
|  | 47659 | 153 | Human adenovirus 2 | AC_000007.1 | 148 | 100.00 |
|  | 5319 | 2557 | Peridroma alphabaculovirus | NC_024625.1 | 139 | 76.98 |
|  | 32524 | 205 | Pseudomonas phage phiKZ | NC_004629.1 | 111 | 82.88 |
|  | 7544 | 1925 | Tokyovirus A1 | NC_030230.1 | 110 | 80.00 |
|  | 19912 | 423 | Escherichia phage JLK-2012 | NC_049942.1 | 98 | 87.76 |
|  | 441 | 7396 | Choristoneura fumiferana granulovirus | NC_008168.1 | 95 | 92.63 |
|  | 19914 | 422 | Salmonella phage SEN8 | NC_047753.1 | 86 | 94.19 |
|  | 22679 | 331 | Aeromonas phage phiAS5 | NC_014636.1 | 85 | 84.71 |
|  | 17695 | 538 | Bacillus phage phIS3501 | NC_019502.1 | 75 | 92.00 |
|  | 38641 | 180 | Yersinia phage fHe-Yen9-04 | NC_042116.1 | 69 | 89.86 |
|  | 5258 | 2583 | Diadromus pulchellus toursvirus | NC_011335.1 | 64 | 90.63 |
|  | 27071 | 253 | Burkholderia phage BcepSaruman | NC_049850.1 | 64 | 92.19 |
|  | 19674 | 433 | Bacillus phage Mater | NC_027366.1 | 62 | 93.55 |
|  | 19040 | 463 | Erwinia phage phiEaH2 | NC_019929.1 | 57 | 92.98 |
|  | 26787 | 256 | Pseudomonas phage PaBG | NC_022096.1 | 57 | 91.23 |
|  | 34184 | 195 | Shigella virus 2019SD1 | NC_049820.1 | 46 | 93.48 |
|  | 5013 | 2676 | Prochlorococcus phage P-SSM2 | NC_006883.2 | 45 | 88.89 |
|  | 13792 | 869 | Pandoravirus dulcis | NC_021858.1 | 44 | 93.18 |
|  | 42004 | 172 | Escherichia phage Lambda_ev099 | NC_049953.1 | 44 | 100.00 |
|  | 225 | 8650 | Cyprinid herpesvirus 2 | NC_019495.1 | 43 | 90.70 |
|  | 17243 | 568 | Enterobacteria phage P2 | NC_041848.1 | 43 | 95.35 |
|  | 2267 | 4200 | Acinetobacter phage | NC_041866.1 | 41 | 95.12 |
|  | 694 | 6523 | Gordonia phage Adgers | NC_048695.1 | 39 | 94.87 |
|  | 14904 | 755 | Human herpesvirus 5 | NC_006273.2 | 38 | 94.74 |
|  | 2751 | 3837 | Cynomolgus cytomegalovirus | NC_033176.1 | 38 | 94.74 |
|  | 3275 | 3496 | Red-crowned crane parvovirus | NC_040603.1 | 35 | 97.14 |
|  | 16110 | 650 | Pandoravirus neocaledonia | NC_037666.1 | 33 | 96.97 |
|  | 19432 | 443 | Mycobacterium phage Wonder | NC_042058.1 | 32 | 96.88 |
|  | 21665 | 360 | Pseudomonas phage SM1 | NC_041877.1 | 31 | 100.00 |
|  | 381 | 7661 | Cydia pomonella granulovirus | NC_002816.1 | 28 | 100.00 |
|  | 14461 | 797 | Gallid alphaherpesvirus 1 | NC_006623.1 | 28 | 100.00 |
|  | 6908 | 2090 | Trichoplusia ni single nucleopolyhedrovirus | NC_007383.1 | 28 | 100.00 |
|  | 2638 | 3916 | Leucania separata nuclear polyhedrosis virus | NC_008348.1 | 28 | 100.00 |
|  | 42523 | 171 | Mycobacterium phage Myrna | NC_011273.1 | 28 | 100.00 |
|  | 7215 | 2011 | Rabbit associated gemykroznavirus 1 | NC_025729.1 | 28 | 100.00 |
|  | 3768 | 3205 | Chrysochromulina ericina virus | NC_028094.1 | 28 | 100.00 |
|  | 8350 | 1743 | Elephant endotheliotropic herpesvirus 4 | NC_028379.1 | 28 | 100.00 |
|  | 9495 | 1507 | Mycobacterium phage Luchador | NC_028849.1 | 28 | 100.00 |
|  | 17732 | 535 | Pandoravirus macleodensis | NC_037665.1 | 28 | 100.00 |
|  | 3228 | 3528 | Mythimna unipuncta nucleopolyhedrovirus | NC_043530.1 | 28 | 100.00 |
|  | 13238 | 934 | Bacillus phage vB_BthP-Goe4 | NC_049966.1 | 28 | 100.00 |
| Trinity | 4597 | 7558 | Nora virus | NC_007919.3 | 7546 | 97.14 |
|  | 1955 | 1679 | Lymantria dispar multiple nucleopolyhedrovirus | NC_001973.1 | 927 | 75.73 |
|  | 5126 | 690 | Escherichia phage RCS47 | NC_042128.1 | 353 | 89.24 |
|  | 23600 | 229 | Shigella phage Sf22 | NC_042039.1 | 181 | 86.19 |
|  | 2005 | 2574 | Peridroma alphabaculovirus | NC_024625.1 | 130 | 77.69 |
|  | 28235 | 501 | Salmonella phage SEN8 | NC_047753.1 | 112 | 88.39 |
|  | 21823 | 205 | Pseudomonas phage phiKZ | NC_004629.1 | 111 | 82.88 |
|  | 12233 | 2900 | Tokyovirus A1 | NC_030230.1 | 110 | 80.00 |
|  | 26009 | 1600 | Escherichia phage JLK-2012 | NC_049942.1 | 98 | 87.76 |
|  | 1082 | 7396 | Choristoneura fumiferana granulovirus | NC_008168.1 | 95 | 92.63 |
|  | 4848 | 645 | Aeromonas phage phiAS5 | NC_014636.1 | 85 | 84.71 |
|  | 5470 | 1050 | Pseudomonas phage PhiPA3 | NC_028999.1 | 81 | 83.95 |
|  | 4338 | 462 | Bacillus phage phIS3501 | NC_019502.1 | 75 | 92.00 |
|  | 27434 | 2583 | Diadromus pulchellus toursvirus | NC_011335.1 | 64 | 90.63 |
|  | 1472 | 7041 | Prochlorococcus phage P-SSM2 | NC_006883.2 | 45 | 88.89 |
|  | 4517 | 2220 | Pandoravirus dulcis | NC_021858.1 | 44 | 93.18 |
|  | 864 | 4101 | Cyprinid herpesvirus 2 | NC_019495.1 | 43 | 90.70 |
|  | 5397 | 3575 | Cotesia congregata bracovirus | NC_006659.1 | 39 | 97.44 |
|  | 23775 | 310 | Aeribacillus phage AP45 | NC_048651.1 | 39 | 100.00 |
|  | 6537 | 6333 | Gordonia phage Adgers | NC_048695.1 | 39 | 94.87 |
|  | 5174 | 5002 | Cynomolgus cytomegalovirus | NC_033176.1 | 38 | 94.74 |
|  | 2246 | 5749 | Acinetobacter phage YMC11/11/R3177 | NC_041866.1 | 38 | 97.37 |
|  | 28561 | 568 | Enterobacteria phage P2 | NC_041848.1 | 36 | 100.00 |
|  | 2889 | 2546 | Red-crowned crane parvovirus | NC_040603.1 | 35 | 97.14 |
|  | 21142 | 397 | Pandoravirus neocaledonia | NC_037666.1 | 33 | 96.97 |
|  | 29092 | 360 | Pseudomonas phage SM1 | NC_041877.1 | 31 | 100.00 |
|  | 0 | 1289 | Human herpesvirus 5 | NC_006273.2 | 29 | 100.00 |
|  | 3220 | 755 | Cydia pomonella granulovirus | NC_002816.1 | 28 | 100.00 |
|  | 2887 | 689 | Gallid alphaherpesvirus 1 | NC_006623.1 | 28 | 100.00 |
|  | 13375 | 4275 | Trichoplusia ni single nucleopolyhedrovirus | NC_007383.1 | 28 | 100.00 |
|  | 3007 | 599 | Rabbit associated gemykroznavirus 1 | NC_025729.1 | 28 | 100.00 |
|  | 6850 | 1879 | Chrysochromulina ericina virus | NC_028094.1 | 28 | 100.00 |
|  | 2900 | 768 | Elephant endotheliotropic herpesvirus 4 | NC_028379.1 | 28 | 100.00 |
|  | 756 | 2701 | Mycobacterium phage Luchador | NC_028849.1 | 28 | 100.00 |
|  | 5486 | 3543 | Mythimna unipuncta nucleopolyhedrovirus | NC_043530.1 | 28 | 100.00 |
|  | 8292 | 735 | Bacillus phage vB_BthP-Goe4 | NC_049966.1 | 28 | 100.00 |
