# Supplementary material for: CStone: A de novo transcriptome assembler for short-read data that identifies non-chimeric contigs based on underlying graph structure
Source: PLoS Comput Biol. 2021 Nov 23;17(11):e1009631. doi: 10.1371/journal.pcbi.1009631 (PMC8651127; doi:10.1371/journal.pcbi.1009631)
Supplement: S2 Table — (DOCX) [file pcbi.1009631.s006.docx]

**S2 Tabe: Virus reference genomes from NCBI that matched with contigs representing whole-adult 2 using megablast.**

| **Assembler** | **Contig** | **Contig Length** | **Matched Species** | **Accession** | **Length Of Aligned Region** | **Percent Identity** |
| --- | --- | --- | --- | --- | --- | --- |
| CStone | 18809 | 1530 | Lymantria dispar multiple nucleopolyhedrovirus | NC_001973.1 | 911 | 75.85 |
|  | 51611 | 309 | Peridroma alphabaculovirus | NC_024625.1 | 130 | 77.69 |
|  | 49022 | 359 | Tokyovirus A1 | NC_030230.1 | 110 | 80.00 |
|  | 32930 | 348 | Choristoneura fumiferana granulovirus | NC_008168.1 | 77 | 89.61 |
|  | 2463 | 1794 | Diadromus pulchellus toursvirus | NC_011335.1 | 64 | 90.63 |
|  | 617 | 335 | Mycobacterium phage Wonder | NC_042058.1 | 51 | 90.20 |
|  | 56871 | 406 | Mythimna unipuncta nucleopolyhedrovirus | NC_043530.1 | 43 | 88.37 |
|  | 34827 | 684 | Cyprinid herpesvirus 2 | NC_019495.1 | 41 | 90.24 |
|  | 35428 | 1233 | Gordonia phage Adgers | NC_048695.1 | 39 | 94.87 |
|  | 60681 | 205 | Cynomolgus cytomegalovirus | NC_033176.1 | 38 | 94.74 |
|  | 50367 | 493 | Pandoravirus salinus | NC_022098.1 | 31 | 96.77 |
|  | 59418 | 255 | Prochlorococcus phage P-SSM2 | NC_006883.2 | 29 | 100.00 |
|  | 5999 | 2139 | Murid herpesvirus 1 | NC_004065.1 | 28 | 100.00 |
|  | 32066 | 327 | Adoxophyes honmai nucleopolyhedrovirus | NC_004690.1 | 28 | 100.00 |
|  | 32890 | 313 | Agrotis segetum nucleopolyhedrovirus | NC_007921.1 | 28 | 100.00 |
|  | 16067 | 469 | Chrysochromulina ericina virus | NC_028094.1 | 28 | 100.00 |
|  | 49865 | 323 | Mycobacterium phage Luchador | NC_028849.1 | 28 | 100.00 |
| rnaSPAdes | 9640 | 1512 | Lymantria dispar multiple nucleopolyhedrovirus | NC_001973.1 | 927 | 75.73 |
|  | 24827 | 300 | Proteus phage VB_PmiS-Isfahan | NC_041925.1 | 300 | 100.00 |
|  | 27084 | 264 | Human adenovirus 2 | AC_000007.1 | 264 | 100.00 |
|  | 28353 | 247 | Enterobacteria phage O276 | NC_049951.1 | 247 | 100.00 |
|  | 322 | 7753 | Peridroma alphabaculovirus | NC_024625.1 | 139 | 76.98 |
|  | 4752 | 2776 | Tokyovirus A1 | NC_030230.1 | 110 | 80.00 |
|  | 474 | 7124 | Choristoneura fumiferana granulovirus | NC_008168.1 | 95 | 92.63 |
|  | 14843 | 795 | Shamonda orthobunyaviru | NC_018464.1 | 91 | 92.31 |
|  | 5373 | 2548 | Diadromus pulchellus toursvirus | NC_011335.1 | 64 | 90.63 |
|  | 569 | 6822 | Pandoravirus dulcis | NC_021858.1 | 44 | 93.18 |
|  | 6626 | 2169 | Acinetobacter phage YMC11/11/R3177 | NC_041866.1 | 44 | 90.91 |
|  | 615 | 6665 | Cyprinid herpesvirus 2 | NC_019495.1 | 43 | 90.70 |
|  | 3232 | 3504 | Mythimna unipuncta nucleopolyhedrovirus | NC_043530.1 | 43 | 88.37 |
|  | 27703 | 255 | Aeribacillus phage AP45 | NC_048651.1 | 42 | 100.00 |
|  | 15362 | 749 | Human herpesvirus 5 | NC_006273.2 | 41 | 92.68 |
|  | 648 | 6562 | Gordonia phage Adgers | NC_048695.1 | 39 | 94.87 |
|  | 2628 | 3925 | Cynomolgus cytomegalovirus | NC_033176.1 | 38 | 94.74 |
|  | 3249 | 3494 | Red-crowned crane parvovirus | NC_040603.1 | 35 | 97.14 |
|  | 36677 | 187 | Cydia pomonella granulovirus | NC_002816.1 | 34 | 97.06 |
|  | 40709 | 175 | Pandoravirus neocaledonia | NC_037666.1 | 33 | 96.97 |
|  | 42133 | 170 | Cotesia congregata bracovirus | NC_006659.1 | 32 | 100.00 |
|  | 7513 | 1949 | Prochlorococcus phage P-SSM2 | NC_006883.2 | 29 | 100.00 |
|  | 8096 | 1817 | Trichoplusia ni single nucleopolyhedrovirus | NC_007383.1 | 29 | 100.00 |
|  | 30815 | 223 | Rhinolophus associated gemykibivirus 2 | NC_038500.1 | 29 | 100.00 |
|  | 31368 | 218 | Mycobacterium phage Wonder | NC_042058.1 | 29 | 100.00 |
|  | 492 | 7060 | Leucania separata nuclear polyhedrosis virus | NC_008348.1 | 28 | 100.00 |
|  | 13039 | 995 | Enterobacteria phage GEC-3S | NC_025425.1 | 28 | 100.00 |
|  | 10391 | 1381 | Rabbit associated gemykroznavirus 1 | NC_025729.1 | 28 | 100.00 |
|  | 3419 | 3388 | Chrysochromulina ericina virus | NC_028094.1 | 28 | 100.00 |
|  | 1480 | 4991 | Elephant endotheliotropic herpesvirus 4 | NC_028379.1 | 28 | 100.00 |
|  | 9334 | 1572 | Mycobacterium phage Luchador | NC_028849.1 | 28 | 100.00 |
|  | 16250 | 675 | Pandoravirus macleodensis | NC_037665.1 | 28 | 100.00 |
|  | 5660 | 2453 | Oxyplax ochracea nucleopolyhedrovirus | NC_043529.1 | 28 | 100.00 |
|  | 1457 | 5018 | Bacillus phage vB_BthP-Goe4 | NC_049966.1 | 28 | 100.00 |
| Trinity | 25640 | 1616 | Lymantria dispar multiple nucleopolyhedrovirus | NC_001973.1 | 911 | 75.85 |
|  | 27877 | 300 | Proteus phage VB_PmiS-Isfahan | NC_041925.1 | 300 | 100.00 |
|  | 24678 | 247 | Enterobacteria phage O276 | NC_049951.1 | 247 | 100.00 |
|  | 4984 | 4017 | Peridroma alphabaculovirus | NC_024625.1 | 130 | 77.69 |
|  | 16033 | 2776 | Tokyovirus A1 | NC_030230.1 | 110 | 80.00 |
|  | 2418 | 415 | Shamonda orthobunyavirus | NC_018464.1 | 91 | 92.31 |
|  | 23776 | 2548 | Diadromus pulchellus toursvirus | NC_011335.1 | 64 | 90.63 |
|  | 27379 | 455 | ycobacterium phage Wonder | NC_042058.1 | 45 | 88.89 |
|  | 3239 | 4965 | Pandoravirus dulcis | NC_021858.1 | 44 | 93.18 |
|  | 4882 | 2857 | Acinetobacter phage YMC11/11/R3177 | NC_041866.1 | 44 | 90.91 |
|  | 221 | 926 | Cyprinid herpesvirus 2 | NC_019495.1 | 43 | 90.70 |
|  | 5727 | 3517 | Mythimna unipuncta nucleopolyhedrovirus | NC_043530.1 | 43 | 88.37 |
|  | 2390 | 4556 | Simbu orthobunyavirus | NC_018476.1 | 41 | 100.00 |
|  | 927 | 6534 | Gordonia phage Adgers | NC_048695.1 | 39 | 94.87 |
|  | 8851 | 4225 | Cynomolgus cytomegalovirus | NC_033176.1 | 38 | 94.74 |
|  | 13830 | 459 | Trichoplusia ni single nucleopolyhedrovirus | NC_007383.1 | 36 | 94.44 |
|  | 3674 | 2921 | Red-crowned crane parvovirus | NC_040603.1 | 35 | 97.14 |
|  | 16273 | 204 | Mollivirus sibericum | NC_027867.1 | 30 | 100.00 |
|  | 5 | 1275 | Human herpesvirus 5 | NC_006273.2 | 29 | 100.00 |
|  | 1771 | 4219 | Prochlorococcus phage P-SSM2 | NC_006883.2 | 29 | 100.00 |
|  | 2459 | 1267 | Cydia pomonella granulovirus | NC_002816.1 | 28 | 100.00 |
|  | 1298 | 1351 | Leucania separata nuclear polyhedrosis virus | NC_008348.1 | 28 | 100.00 |
|  | 1588 | 1995 | Rabbit associated gemykroznavirus 1 | NC_025729.1 | 28 | 100.00 |
|  | 4697 | 4026 | Chrysochromulina ericina virus | NC_028094.1 | 28 | 100.00 |
|  | 5795 | 900 | Elephant endotheliotropic herpesvirus 4 | NC_028379.1 | 28 | 100.00 |
|  | 3212 | 1572 | Mycobacterium phage Luchador | NC_028849.1 | 28 | 100.00 |
|  | 8133 | 738 | Pandoravirus macleodensis | NC_037665.1 | 28 | 100.00 |
|  | 4354 | 1590 | Bacillus phage vB_BthP-Goe4 | NC_049966.1 | 28 | 100.00 |
