# Supplementary material for: CStone: A de novo transcriptome assembler for short-read data that identifies non-chimeric contigs based on underlying graph structure
Source: PLoS Comput Biol. 2021 Nov 23;17(11):e1009631. doi: 10.1371/journal.pcbi.1009631 (PMC8651127; doi:10.1371/journal.pcbi.1009631)
Supplement: S1 Method — (DOCX) [file pcbi.1009631.s007.docx]

**S1 Method: Demonstrating the effects of chimerism on differential expression analysis.** This experiment was set up to investigate the effects of chimerism within a reference set on the identification of differentially expressed transcripts.

(All reference sets and corresponding datasets are available on the Zendo repository and are associated with the doi number 10.5281/zenodo.5589427 [71]).

**S1 Method**

(1) From the 26,679 cDNA reference transcripts obtained from Ensembl, described for *D. melanogaster* (see table 1), five read datasets, each containing two million read pairs, were simulated. For each, low level read count variation was introduced by allowing the number of reads required for an even coverage, as described in the main manuscript, to be increased by a factor of between 0 and 0.2. The expected per site coverage provided by 2 million reads pairs across the 26,679 transcripts was slightly greater than 7X. These five replicates were allocated to condition A.

(2) A further five replicates were simulated, where this time 1000 of the reference transcripts were selected at random for possible over-expression. For these 1000 genes the number of reads required was increased above the background variation by a factor of between 1 and 5. These five replicates were allocated to condition B.

(3) Each replicate within condition A and B was mapped, using bowtie2 [64], to the cDNA reference templates from they had been simulated, after which the pileup script, of the bbmap package [65], was used to obtain read counts associated with each transcript.

(4) Using these read counts, DESeq2 [49] was run to obtain a list of overly expressed transcripts between conditions A and B (cutoff: p-adj <=0.05). These were transcripts identified as being over expressed between the two conditions in the absence of chimeras.

(5) Ten additional transcript reference sets were created from the 26,679 transcript reference sequences used in (1). Each of these ten, contained 26,679 transcripts, but with an incrementing proportion of these being chimeric forms. The increments used where 5 to 50% in steps of 5. During each increment, the specified percent of transcripts for that increment were randomly selected for modification in a chimeric manner. One of three types of chimera modification was introduced: (i) erroneously swapping parts of expressed transcripts with others (windows between 1 and 5, of between 100 to 250 in length), (ii) introducing regions of sequencing variation (windows between 1 and 5, of between 100 to 250 in length) and (iii) over extension of contigs (by an amount between 100 nt and the length of a randomly selected transcript to be used for over extension).

(6) Steps (3) and (4) where repeated independently for each the ten reference sets created in (5), in conjunction with the read data simulated in (1) and (2); the latter still reflecting conditions A and B. Each repeat produced a list of differentially expressed transcripts that could be associated with the specific level of chimerism present within the reference set used.

(7) Each list of differentially expressed transcripts obtained in (6) was compared back to the list of differentially expressed genes using the non-chimeric reference set (from the initial run of step 4) so that alterations in the detection of differentially expressed transcripts, relative to the extent of chimerism within the reference set used, could be visualized and quantified.
