# Supplementary material for: CStone: A de novo transcriptome assembler for short-read data that identifies non-chimeric contigs based on underlying graph structure
Source: PLoS Comput Biol. 2021 Nov 23;17(11):e1009631. doi: 10.1371/journal.pcbi.1009631 (PMC8651127; doi:10.1371/journal.pcbi.1009631)
Supplement: Attachment — Submitted filename: response_to_reviewers.docx [file pcbi.1009631.s008.docx]

**Response to Reviewer #1**

Linheiro and John Archer presented a computational approach to de novo transcriptome assembly with an emphasis on identifying chimeric contigs. The concept is important for the field and the paper was written clearly. CStone seems to be easily implemented across operating systems, and can also be incorporated into existing tools. But the concept needs to be developed further to warrant publication in PLoS Computational Biology.

We thank reviewer #1 for their time and comments on our manuscript. We are pleased that the reviewer found the area of our work to be interesting, our paper to be written clearly and that the associated software is widely usable and adaptable. We also appreciate each comment and suggestion made in relation to improving the manuscript. Below we indicate the alterations we have made in relation to our responses.

The primary concern with this tool is that ~30% of all contigs assembled were identified as 'chimeric,' which are claimed to affect downstream analysis such as differential gene expression. If this is indeed true and accurate, it would have large ramification for the entire field. If the authors could test out this concept further, how do these chimeric contigs affect differential expression, what's the scope of this issue, how can the identification of the chimeric contigs improve DE analysis, etc., it would help the reviewers to more accurately assess this approach and the conclusion that this tool can 'greatly benefit the user.'

Our aim is to characterize the proportion of contigs that are derived from complex graphs, i.e. those that offer multiple equally viable paths during contig construction; leading to a higher possibility of the contigs being chimeric. The approximate 30% indicated is the percent of contigs outputted that were derived from such complex graphs for the datasets that we used. Depending on the complexity of the transcriptome in question, this value could be higher or lower. We wanted our assembler to give this insight to the user, and we have discussed its relevance more extensively throughout introduction, e.g. lines around 177, as well as the manuscript in general. Another example within the introduction, line 210, is where we have added:

“*Reference contigs labelled in this manner encourage dependent results to be presented with increased objectivity by maintaining the context of complexity, and ambiguity, present during construction. For example, the identification of a differentially expressed gene associated with a level (iii) contig can be considered more speculatively, whilst for a level (i) contig, more certainty can be assumed. Our approach is not solving the problem of de novo assembled chimeras, but it is improving the interface between assembly software and result interpretation.”*

The reviewer specifically asked about how such chimeric content could affect downstream analysis in relation to differential expression. We elaborate on this within paragraph 2 of the introduction, line 179, and we have included an additional study to explicitly demonstrate this. The latter has been added to the end of the “Design and Implementation section” under the heading “(3) Effects of Chimerism on Differential Expression”. The details of the experiment that we implemented are available in Methods S1, and the results are presented within the new Figure 14, where it is evident that as chimerism within the references sequences increases, the outcome of detecting differential expressed transcripts using ten unchanging input read datasets does not remaining constant.

This observation is vitally important in relation to the development of tools for short-read *de novo* assembly, and to the people using them, as dependent results vary in a manner that has been to date poorly quantified. The reviewer is correct in saying that “it would have large ramification for the entire field”, and this is the point that we are trying to highlight, and to improve, though the labelling of the contigs produced by our assembler. In the future, as longer reads become routine, the entire assembly process on short-read data may be avoided. We have included a mention of this within the introduction in response to a comment made by reviewer 2, line 162, but given the amount of such data already generated, it is still imperative to highlight this problem and raise awareness.

how can the identification of the chimeric contigs improve DE analysis?

Still related to above, we are not improving differential analysis so to speak, just allowing the user to keep results, where relevant, in the context of the ambiguities associated with chimerism. This is exceptionally important, as there is a difference in reliability between non-chimeric transcripts identified as being differentially expressed and ones that are chimeric. We have clarified this throughout the manuscript, for example within paragraph 2 of the introduction we discuss the causes and consequences of chimeric content on differential expression analysis, and within the conclusion we state:

*“More importantly, it adds additional information to the output in relation to chimerism that: (i) can benefit the user, and research community as a whole, during the presentation and discussion of results, by maintaining the context of the ambiguities associated with chimerism when relevant and, (ii)”*

Another issue that needs to be addressed is that especially when using real data, contigs generated by CStone have a wider spread towards shorter contigs, compared with Trinity and SPADES (Figure S4).

For the simulated datasets, figure S4 is a visualization of the per cent identities achieved within the aligned regions of contigs and the reference transcript to which they matched. More importantly, table 4 presents a summary of this information where it can be observed that the means achieved by CStone of 99.80%, 99.25, 98.65 and 99.58%, for fruit fly, leopard, rat and canary, as well as the four medians of 100%, do not generally perform worse than those of rnaSPAdes or Trinity. It is true that there is a wider range than for the latter two assemblers, but all points are above 70%, and the majority are above 99% as indicated by means and medians. The points in figure S4 visually emphasize the minority of lower identities achieved but table 4 must be kept in mind when looking at it. We have explicitly mentioned this at line 484. With this in mind our aim was to demonstrate that CStone can produce contigs that are of reasonable quality when compared to those created by current state-of-the-art assemblers, Trinity being arguably the most widely adopted over the last decade, and that these contigs could be used to demonstrate the possibility of adding an indication of whether or not they were derived from graphs with no ambiguous paths, and thus that cannot chimeric. We have emphasised this throughout the manuscript further.

). Also, the contigs generated by CStone seem to have lower percent identity overall (Table 6). Could these factors increase the possibility of identifying chimeric contigs, which could be potential not chimeric?

In relation to the original table 6, we noticed that for the three assemblers the values were identical for the means that were associated to each dataset. This was due to rounding values to just two decimal places. We have now increased this to four decimal places. Within this table the percent identity achieved by CStone does not appear to be lower than that of the other assemblers. What is lower are the values in the “No. of cDNA transcripts matched” column. We have explicitly stated this, line 549, and discussed the reasoning why we feel this is the case; subsequent two paragraphs.

However, more generally in relation to the question “Could these factors increase the possibility of identifying chimeric contig?”. Simple graphs produce one or few, unambiguous choices, complex ones produce more choices with increased ambiguity. These graphs are based on the tried and tested de Bruijn approach, that we discuss in within the introduction, line 192, and for which there are multiple studies analysing their suitability in accommodating the diversity present within biological data. We are maintaining this assumption, but indicating that given such diversity represented on such a graph, if multiple ambiguous paths exist then there is a high possibility that the one selected is chimeric and care should be taken. Based on the reviewers comment we have tried to increase clarity on this throughout the introduction, e.g. line 204.

Minor comments:
Adding line numbers will help the reviewers to navigate the manuscript much more easily.

Added. But those in the track changes document are different from the clean manuscript. We have given the ones from the clean copy.

This might be writing preference, but it would help to sometimes add a comma to aid the reading of the sentences. For example, at the beginning of the third paragraph in Design and Implementation, "For each graph, local cycles ..." would seem more readable. And there are many examples throughout the manuscript. Even if not at all places, some commas would still aid the reading of the sentences, which might otherwise be confusing.

We have proof read the manuscript, multiple times, and in conjunction with comments from reviewer 2, have attempted to improve our readability and usage of punctuation.

Second paragraph of the Introduction, approach's -> approaches,

Altered.

effects -> affects

Paragraph 2 has changed, and comment no longer relevant, but in general from a search on google we found the following: ***“Affect*** *is a verb – “to affect” – meaning to influence or have an impact on something. Effect is the noun – “an effect (a positive or a negative effect) is the result of being affected by something”* Prior to this comment we were using “affect” and “effect” inconsistently and now I think that is improved. Most things we use are in relation to the noun with “the” before it, so in many places we have used “effect”. Also, in the new sub-heading added for reviewer 1, we have “Effects of Chimerism on Differential Expression”. We are under the impression that this is OK, but if the reviewer disagrees we will alter.

**Response to Reviewer** #**2**

De novo transcriptome assembly of short RNA-Seq reads is still a challenging problem although tackled but not fully solved by various methods and tools over the last decade. One particular problem lies in the reconstruction of chimeric contigs representing falsely assembled transcripts. This can happen because reads are first reduced to shorter kmers of specific lengths and put into a graph structure that is then traversed to find connected paths finally representing contigs and thus transcripts. However, junctions in the graph structure can result in chimeric contigs that mix up different transcript sequences that biologically dont belong together.

Here, Raquel Linheiro and John Archer present CStone, a basic de novo assembly tool that utilizes kmers and a de Brujin-like graph structure to not only assemble contigs but to further classify them according to their chimeric potential. Thus, each contig gets categorized based on its original representation in the graph, helping to decide if the contig might be chimeric and thus needs further investigation or should be considered carefully in downstream analyses.

CStone, written in Java, is compared against two other state-of-the-art assemblers on simulated and real RNA-Seq data and shows similar performance but, more importantly, adds the functionality to evaluate the chimeric level of resulting contigs. I think that the presented method to categorize contigs according to their chimeric potential is an interesting and important feature that could be added also to other transcriptome (and genome?) assembly tools to further guide researchers on their way of interpreting assembled contig data.

We thank reviewer #2 for their time and comments on our manuscript. From the above summary the reviewer clearly appreciates the area we are working on and we welcome each comment and suggestion made in relation to improving the manuscript. Below, we indicate the alterations to the manuscript (including that of the initial query about “genome?” assembly which we respond to in relation to “the DNA assembly graph” query below), and software.

The code together with example data and a manual is publicly available on Sourceforge and I was able to execute the tool on a MacBook Air.

This is good to know, much of our development was done on laptops with specifications not dis-similar to a MacBook Air. But in reality we are aiming for CStone to be utilized on higher specification machines as the datasets involved could be quite large. However this is obviously user choice. Why we specified running on higher specification machines is we did not want to be held accountable for problems such as limited memory or increased processing time due to reduced cores and ram.

Below, I have some comments and suggestions. The Figures are generally nice and help to understand the approach, however, the manuscript could need some English language editing to be more precise and clear.

[1] Title … a de novo [transcriptome] assembler … ? In general, you could be more precise about the transcriptomic field your tool is developed for. Also, your paper is about RNA-Seq data and transcriptome de novo assembly and not DNA and genome assembly. While one could assume that based on the abstract and introduction, that might be not always clear to the reader. E.g. consider changing sentences like “A crucial part of the de novo assembly process...” into “A crucial part of the transcriptome de novo assembly process...” Actually, from your sourceforge page this is a good sentence to introduce the tool and its scope that I miss in the abstract/paper: “CStone is a [de novo] short-read assembler for RNA-Seq transcriptomic data based on a de Bruijn like approach.”

The reviewer was absolutely correct here, there was clarity missing in relation to specifically saying that we are dealing with short-read transcriptomic assembly. We have altered the title and made our usage of “short-read transcriptomic data” far more clear throughout. The reviewer’s explicit suggestions given above have been incorporated.

Besides, do you think that your tool can also detect and classify chimeric contigs from a DNA assembly graph? Maybe as an future extension?

This is also relevant in relation to the reviewers initial query on genome assembly. Presently CStone is solely a short-read transcriptome assembler. The primary reason for this is because of the way in which the underlying network structure is utilized for classification. Transcriptome assembly deals with many, relatively small, networks that can be classified as either being one of two simple levels or as complex. For genome assembly networks become much fewer and much larger. Even a fairly simple large network, representing a chromosomal fragment, is likely to possess multiple path choices from one side to the other simply because it is so large; spanning millions of bases rather than hundreds or low thousands. Thus, we would expect to rarely see the class one, and similarly class two, networks that we utilize to call a transcript as non-chimeric.

With the removal of classifications based on network complexity, it could be possible to assemble longer fragments representing chromosomes, but then we would need to incorporate other optimisations in dealing within this task, such as looking at paired reads with very large insert sizes, far longer reads (or a mix of both long and short) as well as dealing with extensive repeating regions, which in transcriptomic assembly we do not encounter. In doing this we probably wouldn’t be bring anything that other genome level assemblers haven’t already explored extensively. We have elaborated a little on this within the introduction, line 155.

Consequently, at the moment we are focused on the problem of chimeras within short-read transcriptome assembly. An area that we would prefer to explore in the future would be to divide our level (iii) category further in order to reflect more subtle probabilities of chimerism. For example, rather than just say that a contig simply comes from a complex network, containing ambiguous paths, we would like to be able to place a probability on each contig that reflects its possibility of being chimeric, given the number and types of paths. We have hinted at this within the “Future Directions” section at the end of the manuscript, line 646.

[2] Language

Please check language carefully, there are several minor things such as
high quality → high-quality
whole body → whole-body
etc..

Altered, and we have gone over the manuscript and improved these.

Please pay attention to commas.

“Over extension of contigs, a form of chimera, has also been shown to have a negative impact on differential expression studies” suggestion →
“Overexpansion/Overextension of contigs, a form of chimera, has also been shown to negatively impact differential expression studies.” I have the feeling that in general writing “overextension” as one word might be better - but I’m not a native speaker.

Also in relation to reviewer ones comments we have improved the situation in relation to commas and grammar. We have opted for overextension as the reviewer suggests.

“ is the arrangement information present”
→ do you mean
“ is the arrangement of information present”

Yes and corrected.

“state-of-the art” → “state-of-the-art”

Corrected.

“For graphs of the third type, CStone similarly to other graph based assemblers, uses the metric of read coverage to aid in path choice, but this does not guarantee complete nonchimerism, although high quality representations of underlying transcripts are achieved.” → suggestion (and as an example, I think your paper could benefit from some general language editing)
“For graphs of the third type, CStone, similar to other graph-based assemblers, uses the metric of read coverage to support path selection, but this does not guarantee complete chimera freedom, although high-quality representations of the underlying transcripts are achieved.”

Altered, but we kept the term “non-chimerism” instead of the suggested “chimera freedom”. The latter could be a little ambiguous suggesting that chimeras are free to do what they want within the data. As with our previous responses to the topics covered in this comment, we agree with the reviewer suggestion, and have concentrated a lot of effort on proof reading the manuscript.

“For each graph local cycles between close neighbouring nodes are removed whilst maintaining non-localized paths between junctions” → “For each graph, local cycles between adjacent nodes are removed, while non-localized paths between junctions (intersections?) are retained.”

Altered to suggestion. We kept the term “junction” though, line 255, as we feel that intersection suggests two, or more, paths crossing, but this might not necessary be the case. For example, one path could branch out in two different directions. Junction possibly describes such branching points more accurately, some of which will also be intersections. More trivially we use the set “J” in diagrams and refer to it in text. One set we designate “I” refers to internal nodes. If we use the term intersection, we would have two node entity types starting with “I” and would need to pick a random letter to represent one of them. Now we have Junction = set “J”, Internal = set “I” and External = set “E” which seems convenient.

“During merger...” ? During the merge process? What does “merger” mean? Please check the paragraph.

We opted for the suggested “During the merge process, … ” In relation to term “merger”, a google’s hit says: “*…. A merger is the voluntary fusion of two companies on broadly equal terms into one new legal entity….”* Thus, maybe the term “merger” was inappropriate as it is more of a corporate meaning.

“ For a given graph following refinement, figure 1, on any partial path, before, after or between neighbouring junction nodes, with the exception of external nodes, each node will have two outgoing edges that link to its nearest neighbours, one parent (incoming) and one descendent (outgoing), depending on the direction of traversal.”
→ a complicated sentence, hard to follow. But your Figures are really nice and much easier to understand than the text description. Suggestion: reference your figures more often explaining the graph structure and classification process.

Agreed. It took us a reasonable amount of time to put some sentences together like the example given, in terms of making sure they accurately reflect what we were doing, and yes, the diagrams are clearer. To improve this situation we have tried to ensure usage of commas and grammar is optimized throughout.

“ thus talking a small step” → “ thus taking a small step”

Altered.

“ established assemblers could incorporated” → “ established assemblers can incorporate”

Altered.

“CStone derived its title after this node.” → “CStone got its title based on this node.”

Altered.

Figure 2 caption: please check the caption, the sentence is hard to follow/unclear language.

Updated.

“Trimomatic” → “Trimmomatic”

Altered.

“above 5000 in length” → “above 5000 nt in length”, in general add values

Altered throughout.

[3] Be more precise about DNA or RNA. For example from the abstract: “For real data two datasets, each consisting of ≈30 million read pairs, representing two adult D. melanogaster whole body samples were used.” I guess you mean “whole-body transcriptome samples”?

Yes, we have specified more often that we are using RNA-Seq data, short-read data and transcriptomic data within the abstract as well as throughout the manuscript.

[4] “Where no reference exists reads can be assembled de novo to create one. “
This is not precise. When no genome reference exists you can also not use RNA-Seq reads to assemble one de novo. Or you mean “Where no transcriptome reference exists, ...” Or maybe better rephrase:
“Where no genome reference exists, RNA-Seq reads can be still assembled de novo to create transcripts. For this specific task, many assemblers are available including...”

Generally, as suggested by the reviewer, we have become more specific in our descriptions by using the “RNA-Seq” and “transcriptomic” more frequently; also in order to distinguish more from genomic assemblers. Another example of being more precise in explaining that we are dealing with transcriptomic assembly in is around line 155: *“….. Differences such as these have lead to a distinction in how algorithms are optimized for either genomic or transcriptomic assembly…..“*

[5] “In addition, hybrid approaches that rely on the existence of a reference genome ...” I think “hybrid approach” is not a good term here because it’s mainly understood in the community when you combine short- and long-read data for assembly. Also, better than citing TopHat2 (or mapping tools in general) here you could mention tools such as Stringtie2 that actually perform reference-based transcript assembly.

We have expanded introduction to incorporate the reviewers suggestion, and we explicitly mention Stringtie2, line 159, which is a very valid point. The use of such long reads has potential to dramatically reduce problems of chimeras when they become routine within transcriptomic studies, and we have mentioned this within the introduction, line 162, and more extensively within the “Design and Implementation” section, line 310.

We have also kept our reference to TopHat2, but specified more clearly that they are part of larger pipelines that use genomic references in order to “splice” map RNA-Seq reads and estimate exons, splice-sites and subsequent transcripts, line 165. This is also a valid approach, albeit maybe going out of date, that we feel is worth mentioning in order to emphasis the variety of approaches taken.

[6] short vs long-read transcriptome assembly and impact on chimeric contigs
In the whole manuscript, you never mention that CStone was developed (I assume) for the assembly of short-read RNA-Seq data. I think that’s important having long-read technologies on the rise that might solve such chimeric problems due to short kmers quite easily (“no assembly required”). Of course, your method is still important because of the vast amount of short-read RNA-Seq data out there. Nevertheless, mentioning the impact of longer reads potentially representing full transcript isoforms could be mentioned as well.

Yes, this is a very valid point, and we have clarified that CStone is a short-read assembler throughout. We have also stated that the longer reads in the future will likely dramatically reduce the problem of chimeras, lines 162 and 310 (related paragraph).

[7] kmer length

You are using a fixed kmer size of 40 for CStone. Is it possible to use other kmer sizes? Did you test other sizes? Edit: I just read at Sourceforge that you actually can adjust the kmer size, so you could mention that briefly in the manuscript as well.

We now mention within the manuscript that kmer size can be adjusted, line 244. The QuickStart.pdf file, and sourceforge wiki, have also been updated. In terms of our “k” parameter, we selected a length of 40 nt as default, as this is long enough to allow for a good sequence signal in relation to connecting edges reliably. This is related to figure S1, where it is seen that kmers above 20 nt in length are generally good at representing the diversity found within the data. Additionally, a value of 40 nt lies between the two established assemblers that we were testing against. We now explicitly mentioned this within the graph construction section, line 243.

We did not extensively test other kmer sizes, and such an exploration will inevitably result in subtle variations between assemblies that are dependent on transcriptome complexity, read cover, gene expression levels and many other factors. This will hold true for any assembler tested. Effectively we wanted to select a safe parameter, in the middle of two established assemblers, that did not show any divergent behaviour (figure S1) and that could be a good default parameter. Exploring the subtle relationship between kmer size, transcriptome complexity, assembly success and the range of other factors involved, across multiple assemblers, would be a very nice study, within the area of kmer optimization, but we felt that at this time it was beyond what we were hoping to achieve with this manuscript. Exploring it just for our software, on one or two factors, and limited data, would not add practical usability, and the subtle differences in effects are quite open ended; but are also documented and studied separately, hinted at around line 193. In order not to enter this realm of kmer optimization we selected 40 nt for the reasons described in the first paragraph of this point.

[8] Figure 3: “In panel (c) one path (most likely chimeric), of the possible 12, is shown (inset).”
On what is your assumption based that this path likely results in a chimeric contig? Because the number of possible paths traversing the cornerstone node is high?

We understand why the reviewer asked this question and have altered the legend to include: “In panel (c) one path (inset), of the possible 12, is shown. In the absence of read coverage information, as in this example, the path is most likely chimeric as it turns away from nearest exit node and follows a more winding route. However, within real graphs coverage information could provide a justification for such a route.”

[9] Reconstruction of lowly expressed transcripts: “For levels 2 and 3 the first 10 paths from each E1 starting node, level 2 only having one node within E1, are sorted by mean read coverage and the top three are used to construct contigs in a similar manner to that done for level 1.” → Does this mean that your approach might miss transcripts that are lowly expressed? E.g. a specific transcript isoform that is just rare and thus lower expressed as others?

We have separated the previous internal parameter, that specifies the maximum number of contigs outputted per graph, into two separate externally available ones that the user can alter. The first “–ps”, is the number of contigs produced from simple graphs, and the second is “–pc” which is the number of contigs produced from complex graphs. The quick_start.pdf, and sourceforge wiki, have been updated with this information. However, this comment touches an issue with all short-read graph based assemblers and at line 306 we have added the following “note” outlining the limitations, but that we hope the reviewer can accept.

*“Specifying the number of contigs per graph is necessary as it is not feasible to output all “possibly viable” paths from every graph. For some graphs there could be tens to hundreds of paths. We opted for outputting the top three best paths, based on maximized read coverage, in order to provide a reliable representation of gene families; in a way that could provide insight to contig non-chimerism. This covers many bioinformatic needs, including: (i) that of obtaining reliable references from gene families, where information on chimerism is relevant, such as when looking at co-evolving sites, geno-to-pheno altering polymorphisms or recombinant breakpoint and (ii) many differential expression experiments where the advantage of having information on the reliability of read counts, see method S1 from subsection (3), out-weighs that of obtaining large lists of differentially expressed transcripts based on annotating and mapping to potentially hundreds of thousands of poorly supported contigs. However, the approach of limiting paths is not optimal if attempting to characterize all “true” isoforms from highly complex families; although given the advent of long read sequencing technologies, short-read data would be a poor choice for this.*”

We placed this note next to where we mention the parameter within the results section, as that is where we think it is most appropriate.

As an extra note for the reviewer, if for example, a short-read transcriptome assembler took a contig from every path, within every graph, millions of contigs would be possible, many of which would annotate as they share sequence similarity with the reads that they were constructed from, which in turn shares similarity to expressed genes. This is effectively what all short-read graph based assemblers “could” do. But to attempt to be more “correct” the best paths are then selected, usually based on read cover. However, all paths will be covered by something as the parts of the network that they represent had to be derived from reads sequenced from a gene. It is a very difficult line to draw in terms of determining for each individual network how deep can you go before you are effectively creating purely chimeric “junk”, i.e. not even well supported chimeras (well supported being based on high read cover) that closely reflect transcripts. With this in mind, we decided on a flat limit of the top three based on read cover.

This is the issue that hopefully long reads will solve; that the reviewer has pointed out earlier. Within benchmarking studies the performance of differing tools are tusually ested against each other, but this does usually not highlight the underlying problem with short-read assembly; while it does give many users over-confidence that what they are selecting a correct approach if they apply a tool that outperforms others on the metric used in a relative manner. Figure 9 and 13 in this manuscript show, that for the three assemblers presented in this manuscript, there is at least good general agreeability. Here we are effectively saying that we are not solving the general short-read assembler problem, but rather we are highlighting it and attempting to give the user some intuitive access to the contigs in the context of the problem; while at the same time outputting what we believe are the most reliable contigs from each family present.

[10] “Reads were assembled using CStone, Trinity, and rnaSPAdes”. Parameters and version numbers for Trinity and rnaSPAdes are missing.

These have been added to the Demonstration section under the “Design and Implementation” heading within the manuscript, line 358. This was an oversight by us, but in both cases the latest versions were used. We mention the versions once in the first use and after that we just refer to the names.

[11] Windows and Ubuntu specs: I think it’s enough to have the detailed specifications written once (either in the text or Table 2 caption).

Agreed, we kept the specifications within the figure legend.

[12] Can you somehow distinguish a) chimeric contigs due to mis-assembly and b) chimeric contigs that are actually derived from alternative splicing? For example, roughly one-third of your contigs from simulated data fall into category 3 that indicates chimeric contigs (Figure 6). Thus, if I get this information now from CStone I can still not tell if these are “real” transcripts or assembly artifacts, or? Or is the goal mainly to distinguish contigs that are clearly non-chimeric (categories 1 and 2)?

OK, this is the latter, i.e. we are saying that “the goal is to distinguish contigs that are clearly non-chimeric (levels 1 and 2).”, based on the already existing context of the underlying graph. Anything within the level (iii) category may, or may not, be a non-chimeric contig. If it is not, as with other short-read assemblers, it will still be guaranteed to be a close representative, as paths are guided by the highest read coverage (but can be chimeric) and we pick the top 3 (see extensive response to reviewer point 9).

Basically, our system is designed to say that if something comes from a simple graph, then it can be guaranteed to be non-chimeric, based on the already validated ability of de Bruijn graphs to structurally represent biological data. If something comes from a level three graph, we cannot improve on knowing its level of chimerism, if any. We are not really doing anything new, just adding the information about the context of the graph that each contig was derived from. We specifically state this at line 204. This information already exists once a graph has been assembled; we just output it along with the contigs; having 3 classification levels for graphs, two simple types with no ambiguity and one complex (everything else). We highlight this further throughout the text that our classification levels are more about reflecting the context of the graph.

[13] “while for Trinity and rnaSPAdes these numbers were 20,628 and 26,446 as well as 19,131 and 20,865 respectively, table 5” → Did you check these exceptionally long contigs for the real data set? Can they be annotated or are they even representing some contamination (virus, ...)?

For whole-adult 1 the number of contigs above 5000 nt was 19, 1,543 and 3,666 for CStone, rnaSPAdes and Trinity respectively, while for whole adult 2 these numbers were 29, 1,471 and 7,642 (Table 5). For Trinity alone 12.7% of contigs from whole-adult 2 were above 5000 nt, the longest being 26,446, which is one of the numbers referred to by the reviewer. In relation to the reviewers comment we have added the following text, line 563, to the manuscript, along with supplementary tables 1 ad 2.

*“To investigate what proportion of contigs greater than 5000 nt in length were due to viral contamination, all viral reference genomes (≈10,000) from NCBI were downloaded (https://www.ncbi.nlm.nih.gov/labs/virus/). For whole-adult 1, out of the 1,543 and 3,666 contigs from rnaSPAdes and Trinity, a single match to a Nora virus genome, identified using megablast, was observed (Table S1). Out of the 19 contigs from CStone no match occurred. For contigs below, or equal to, 5000 nt in length, each assembler produced just three matches, where the length of the matching region was above 200 nt. For CStone two of these being to the Nora virus previously identified. These results indicate that for whole-adult 1 contamination by virus genomes was minimum. For whole-adult 2 a similar outcome was found (Table S2).”*

Additionally we have added the following text at line 573:

*“For contigs greater than 5000 nt in length, the proportion of aligned regions, relative to contig length, are lower and have an increased range (Figure 12). This is likely due to contigs being overly extended relative to their best cDNA reference match and/or having internal regions that do not align. Importantly for all assemblers, contigs below or equal to 5000 nt in length, produced far higher portions of aligned regions indicating completeness relative to matching cDNA references; those from CStone possessing the narrowest range of high values.”*

[14] “Additionally, CStone is small in size with minimum code and no dependencies, simplifying modification and incorporation into other bioinformatics tools, packages and pipelines” → I generally agree but I think CStone written in Java might not be able to be integrated that easily into existing tools that are mainly Python-based? At least, while Java developers will be happy about that others might not :) However, the method idea itself can surely be re-written as an extension for existing and well-established assembly tools. By saying that, I’m afraid that sourceforge might not be the best code base for the idea of presenting code that should be re-used and integrated/adjusted. Most developers are used to GitHub or GitLab where you can also easily write and open issues along with referencing the code and sending merge requests or forking the whole project. Actually, that would really allow re-using your codebase. I’m not so experienced with sourceforge but for me, it seems like the platform is not so developer-friendly. Thus, you might want to consider moving your code to Git{Hub,Lab}? Don’t get me wrong, you already did a great job in documenting your code and writing manuals but I have the feeling your work could benefit from such a transition.

By integration into other tools we meant that if the “other” tool is in Java for example, then the code can: (i) either be directly ported across, or (ii) the jar file itself can be imported, which is preferable. In terms of other languages, such as python, we guessed that the latter situation would apply, as the jar file could be executed directly from within another language without modification. For example, if someone was writing a tool/pileline that needed an assembly, then they could import the cstone.jar file and the only external requirement is the java runtime environment.

However, that said, our approach is pretty straight forward, and it could be re-written completely in a different language. Achieving a glass box approach to the software was one of our aims when writing the manuscript primarily to: (i) provide the user with intuitive insight on how the assembly is working and how it affects data, (ii) potential to be implemented as a learning tool in the future with for example graphical displays of each step, and (iii) general clarity so if someone wanted to start to write their own assembler, even an improved tool, in what ever language they choose, they could still take a lot from looking at work.

In terms of GitHub, yes, it looks great but at the moment the free account allows for 500MB of files to be uploaded with the project, which is not enough for our test data and paper datasets. Sourceforge allowed us to have 2GB limit which was sufficient. Additionally, on Sourceforge it is possible to fork or clone projects, but the developer(s) maintain more control. We expect the majority of our users to use the website to get to the jar file, test data and documentation. In terms of people that would like to take part further with the development, we would happily to add them (if appropriate) to the developer’s team. As an aside note, when we did a quick search for the difference between the two platforms we got:

*“If you want to develop an open source project and you want the project to be an application that targets the end users, SourceForge is the best option to be the repository. The reason is that it is focused on converting your project into an executable file along with an installer so that the end users can install the application and use it daily life. But if you are focusing on creating an open source project where it is going to be a collection of packages, GitHub is ideal for such development. With such a development, you end users are primarily going to be developers such as developing programming languages and frameworks.”*

(https://www.freelancinggig.com/blog/2018/05/24/what-is-the-difference-between-sourceforge-and-github/)

We are kindive leaning towards “end users can install the application and use it daily life” with option to add developers where appropriate.

In summary, we are greatful for the suggestion of GitHub, and it is definitely something we would like to consider for future projects, but for now we hope that the reviewer is OK with the current *SourceForge host*.

[15] Sourceforge manual: “Each time the software is run, this directory will be deleated and recreated, so it is best to copy it somewhere else (or rename it) between usages. This was done to protect the user from accidentally deleting directories.” → I dont agree that this is good behaviour. It would be better if the output directory gets a timestamp or unique name assigned or the user is warned if the output directory already exists instead of just deleting results the user might run hours or days for.

Based on the reviewers comment we have altered this. The user now specifies the output directory with the “-o” parameter. The quick_start.pdf, and sourceforge wiki, that comes with the software, as well as the quick start wiki page, have been updated with this information under “Running CStone” heading.
