# Supplementary material for: CStone: A de novo transcriptome assembler for short-read data that identifies non-chimeric contigs based on underlying graph structure
Source: PLoS Comput Biol. 2021 Nov 23;17(11):e1009631. doi: 10.1371/journal.pcbi.1009631 (PMC8651127; doi:10.1371/journal.pcbi.1009631)
Supplement: Attachment — Submitted filename: response_to_reviewers_revision_2.docx [file pcbi.1009631.s009.docx]

**Response to Reviewer #1**

The authors have addressed my comments and the revised manuscript is deemed adequate for publication.

We thank reviewer 1 for the input previously provided. The comments were valuable for improving our manuscript.

**Response to Reviewer** #**2**

thank you very much for carefully answering my questions and especially for the additional analyses and effort you put into the manuscript. I think the manuscript significantly improved from the first version, not at least due to the language editing and the more precise wording (RNA-Seq, ...). Good job!

We would like to thank reviewer 2 for comments provided. They proved highly valuable in relation to improving the manuscripts content and readability.

I'm also ok with your code living on SourceForge. However, the point that GitHub does not have enough space is not entirely valid for me. Someone that wants to use your code does anyway not expect to download 2GB of data from a repository. Thus, my preferred way would be to put code and light-weight content on a code repository and example data/large files on another resource such as Zenodo or OSF (Open Science Framework). Then, such data resources could be referenced from the code repository. However, I also agree that this is a matter of taste and you justified why you want to keep your code on SourceForge and I'm fine with that.

We are happy that reviewer 2 is OK with us maintaining the current SourceForge repository in relation to distributing our software. We agree with the reviewer that providing the datasets on a different platform to the development repository would be a more pleasant solution. Thus, we have uploaded the datasets to the Zenodo platform as suggested. The doi references for the datasets provided by Zenodo have been included within the paper where appropriate:

Line 340: “These datasets are available on the open-access repository Zendo and are associated with the url https://doi.org/10.5281/zenodo.5589533 [64].”, and line 394: “All reference sets and corresponding datasets are available on the Zendo repository and are associated with the url https://doi.org/10.5281/zenodo.5589427 [71].”

These datasets have been removed from the SourceForge project site. On the SourceForge project site we have maintained the small test dataset (not relevant to paper analysis) that we feel should be available immediately with the software to aid the user in verifying execution. We have clarified this within the Availability section (line 675).

Also, thanks for the detailed explanation regarding the application for DNA graphs. You're right and after your detailed explanation, I think it is good that you clearly focus on this chimeric problem in the context of short-read RNA-Seq data.

We are glad that the reviewer agrees that the scope of this manuscript is in relation to short-read data. Indeed many of the comments provided helped us to clarify this.

Regarding [9] Reconstruction of lowly expressed transcript
Thanks for the explanation, this makes it more clear. I also better understand now why you are limiting the number of contigs derived from the graph and thus focusing on the paths with higher coverage as a priority. However, this still implies that low-coverage transcripts might be just missed by your approach, or? Did you look into that? E.g. in your simulated data did you also had low-coverage contigs and how do rnaSPAdes, Trinity, and CStone perform then? I dont want to produce too much extra work here that might be out of the main scope of the paper, especially bc/ you nicely wrap the topic up via: "Here we are effectively saying that we are not solving thegeneral short-read assembler problem, but ratherwe arehighlighting it and attempting to give the user some intuitive access to the contigs in the context of theproblem;while at the same timeoutputting what we believe are the most reliable contigs from each family present." in your p2p letter. Thus, I'm also fine with a discussion of this topic and making the reader/user aware of this, if further needed.

As long as a gene family is represented by reads, a graph will be constructed and regardless of how low the read coverage is within that graph paths will be outputted. If many paths exist then it is the top three (by default) based on coverage that are used. This parameter can be altered as of the previous suggestion by this reviewer. Thus, our contigs will represent transcripts derived from low covered graphs. To clarify this we have added the following text within the “Design and Implementation**”**at line 325:

“It should be noted here that the default top three paths per graph outputted by CStone are relative to each individual graph created. The manner in which graphs representing gene families are constructed is independent of read cover. As long as a gene family is represented by reads, a graph can be constructed. If on that graph there are many paths, it is the top thee, based on coverage, that are used. Importantly, this means that gene families associated with low expression will still be represented within the output.”

Also in in relation to this we have added the following to the results (line 465):

“For fruit fly, leopard, rat and canary, the overall numbers of contigs produced by CStone fall between those of rnaSPAdes and Trinity, the latter producing the highest numbers. In table 3 it is observed that for CStone contig numbers were 20939 (fruit fly), 29778 (leopard), 26703 (rat) and 21811 (canary). These were produced from 18520, 29465, 25550 and 21517 underlying graphs respectively (figure s2). For Trinity the numbers of contigs created were 24947 (fruit fly), 33709 (leopard), 36327 (rat) and 29399 (canary), and were produced from 15136, 22181, 24077 and 16678 underlying graphs, as derived from the output contig files. These numbers indicate that CStone, although creating fewer contigs relative to Trinity, does not represent fewer networks, where networks are striving to have a one-to-one representation of gene families.”

Besides, this very long and encapsulated paragraph you newly proposed could be simplified bc/ it is hard to follow:

"This covers many bioinformatic needs, including: (i) that of obtaining reliable references from gene families,where information on chimerism is relevant, such as when looking at co-evolvingsites, geno-to-pheno alteringpolymorphisms or recombinant breakpoint and (ii) many differential expression experiments where the advantage of having information on the reliability of read counts, see method S1 from subsection (3), out-weighs that of obtaining large lists of differentially expressed transcripts based on annotating and mapping to potentially hundreds of thousands of poorly supported contigs."

(Line 318) We reworded the above to: “This covers areas of analysis where obtaining reference sequences maintaining exact evolutionary relationships between sites is important, for example, when looking at co-evolving sites, geno-to-pheno altering polymorphisms or recombinant-breakpoints. It also applies to differential experiments where the reliability of read counts at a gene family level out-weighs that of identifying ambiguous isoforms, many of which are artefacts of the short-read assembly graph traversal process. Limiting paths is less optimal if attempting to characterize all “true” isoforms from complex families; although given the advent of long-read sequencing technologies, the sole use of short-read data in conjunction with heuristic short-read *de novo* assemblers should be avoided.”

Also, I think:" ... or recombinant breakpoint[s]..." and "... long-read sequencing " (missing the dash)

Altered. Lines: 315 and 319

also "... short-read data would be a poor choice for this" --> maybe better "... which are anyway difficult to fully reconstruct from short reads alone" (or something like that)

(Line 318) Changed to: “Limiting paths is less optimal if attempting to characterize all “true” isoforms from complex families; although given the advent of long-read sequencing technologies, the sole use of short-read data in conjunction with heuristic short-read *de novo* assemblers should be avoided.

Thank you very much for all the work

It has been a very helpful to work with these constructive and detailed comments and we greatly appreciate the inputs that have made our manuscript far clearer. Thanks.
